# How to Set Up Central Isolation Sites to Prevent Re-outbreaks From Imported Cases of COVID-19: The Experience of Shanghai, China

**DOI:** 10.1017/dmp.2020.429

**Published:** 2020-11-04

**Authors:** Wei Zhang, Zhiru Ge, Jide Lu, Hairong Wang, Yawei Yang

**Affiliations:** 1 Department of Cardiology, Gongli Hospital of Shanghai Pudong New Area, Shanghai, China; 2 Department of Cardiology, Yueyang Hospital of Integrated Traditional Chinese and Western Medicine, Shanghai University of Traditional Chinese medicine, Shanghai, China

**Keywords:** COVID-19, epidemic, medical observation, quarantine

## Abstract

As strict measures were taken, the coronavirus disease 2019 (COVID-19) epidemic has been gradually brought under control. As a port city, Shanghai’s main problem has shifted from treating local cases to preventing foreign imports. To prevent the re-outbreak of COVID-19 caused by imported cases, the Shanghai government has set up central isolation sites for all people entering the country from abroad to be placed under medical observation. This report describes how to set up central isolation sites and run them effectively. We put isolation sites in transformed hotels, arranged personnel according to a huge data network, and set up specific procedures to manage guests. The epidemic situation in Shanghai has confirmed the feasibility and effectiveness of the methods that other jurisdictions can adapt for their use.

**S**hanghai is 1 of China’s economic centers and an international metropolis with a population of more than 24 million. To curb the spread of the coronavirus disease 2019 (COVID-19), the government has taken some measures, such as closing educational institutions, closing shops and restaurants, suspending public gatherings, checking the body temperature of all people entering Shanghai, and home-isolation of all from the key epidemic regions.^1–3^ These measures have achieved good results, and the epidemic situation in Shanghai is not serious. With widespread outbreak outside China, many Chinese and foreigners have returned to Shanghai from overseas since March. Shanghai is under pressure to prevent re-outbreaks from imported cases. Asymptomatic people who returned to Shanghai from affected areas overseas were immediately placed under quarantine conditions and must undergo a 14-d quarantine at home or in designated facilities. There is a large number of people who only transit in Shanghai and do not have the available quarantine conditions at home, or are worried about spreading the virus to their families. To deal with these problems, the government has set up several centralized isolation sites to keep these individuals under medical observation during their quarantine.

As of June 5, 2020, Shanghai has confirmed a total of 336 imported patients with COVID-19 and released 89,062 (26,573 from central isolation sites) from quarantine, which is most likely to prevent the re-outbreak of COVID-19.^4^

## How To Set Up Central Isolation Sites

### Site Selection

All isolation sites were located in hotels. Compared with a makeshift hospital, a hotel has a single room per person that can meet the isolation conditions, which are convenient for providing food and various services, as well as for medical observation. At present, because the disease is spreading widely, tourism is shut down, and many businesses suffered huge losses, including hotels, which were facing cash flow shortage. Some hotel owners are willing to cooperate with other institutions to reduce their losses;thus, the entire hotels were rented at reduced prices.

The hotel should be located as close as possible to the designated COVID-19 treatment hospital or fever clinic while maintaining a reasonable distance from residential areas. The site should be able to deal with emergencies quickly and conveniently while avoiding panic to local residents. If there was a hospital near the airport, a nearby hotel would be the best choice to be an isolation site.

The number of isolation sites should be set dynamically according to the needs for isolation personnel and the reception capacity of each designated hospital. Taking Shanghai as an example, there were more than 40 isolation sites at its peak.

Most hotels can be re-used after upgrading and minor renovations, which were mainly divided into the polluted area, semi-polluted area, and the clean area. The polluted area is an area where personnel receive medical observation, including temporary storage and disposal of items contaminated by their blood, body fluids, secretions, and excreta. It includes ward, filth room, and patient entrance, exit, etc. The semi-polluted area is an area with doors on both sides between the clean area and the polluted area. It is similar to a buffer zone and serves as a preparation room for medical personnel, who often remove their personal protective equipment (PPE) here. The clean area is an area that is not easily contaminated by the patient’s blood, body fluids, pathogenic microorganisms, and other substances in the area for the diagnosis and treatment of respiratory infectious diseases, and should not be entered by the patient with infectious diseases. It includes the duty room, toilet, men’s and women’s changing room, bathroom, and storage room, catering room, etc.^5^

**Figure 1.**
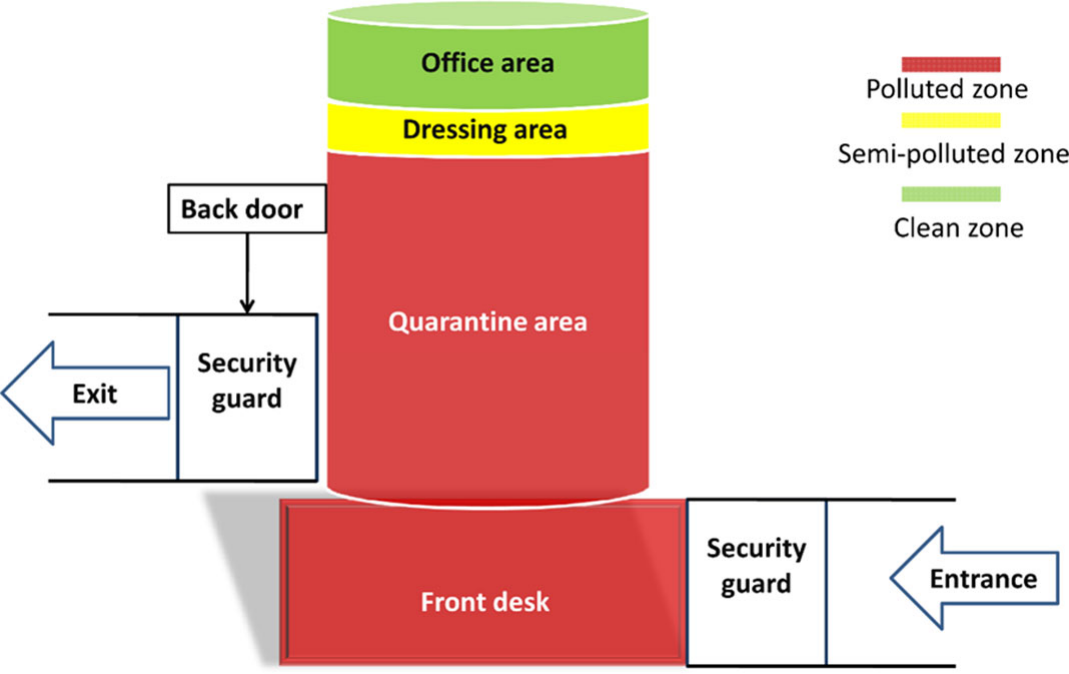
Schematic of hotel upgrades and renovations to establish a central isolation site.

Generally, the lower floors of the hotel can be used as the polluted area for the guests to live in, and 1 floor above the polluted area should be reserved as a semi-polluted area for changing PPE. The upper floors of the hotel should be reserved for offices and for staff living quarters as the clean area. We closed most of the entrances and exits and set up separate entrances and exits for personnel management. Staff access was provided by a separate elevator to the office area. It is worth noting that the fire control measures should be carefully checked, and the fire exits should be divided so that personnel can be quickly evacuated in case of a fire (Figure 1).

### Management Team Composition and Work Division

The management team was divided into 5 taskforces, consisting of medical staff, Centers for Disease Control (CDC) staff, government staff, security staff, and hotel staff. The medical staffs were mobilized from Shanghai municipal hospitals and were mainly responsible for medical observation and nursing. The CDC staff will provide guidance on the transformation of the hotel into 3 zones, personal protection training, and reporting the epidemic data. The government personnel are responsible for coordinating various affairs, such as information sharing between the airport and the isolation sites, arranging personnel entry, preparation of various supplies, coordination of transportation, etc. The security personnel are responsible for the safety of the hotel and its personnel and to prevent other unrelated personnel from straying into the polluted area. The hotel staffs are responsible for providing all kinds of room services, such as food delivery and daily necessities.

The medical staffs were divided into 4 teams, each consists of doctors, nurses, and health-care workers, who worked 6-h shifts per day. According to our experiences, we usually need 1 doctor, 2 nurses, and 1 health-care worker for every 200 guests. All staffs at the isolation site are required to be trained to wear and remove personal protective items before they are accepted.

### Passenger Access

Big-data platforms and information sharing plays a vital role. According to the “*Law of the People’s Republic of China on the prevention and treatment of infectious diseases*”, all units and individuals in China’s territory must accept investigations by disease prevention and control agencies into infectious diseases and provide truthful information. The Institution for Disease Control and Prevention shall not disclose any information or data that may concern personal privacy. The Shanghai government has set up a very large and accurate information sharing network, by interviewing and tracing passengers from the epidemic areas aboard, the network will collect information on passengers’ flight, seat information, destination, travel, and contacts information. This information provided a critical database for tracing close contacts.

During the current situation, at the airport, the required isolation personnel and the remaining room information are shared by the government personnel at each site, and the airport staff will arrange guests to enter the isolation site in a proper manner. All of the guests enter the isolation sites from a dedicated airport bus to avoid contact with other people. After entering the isolation sites, the guests are assigned rooms by adhering to the principle of “1 room per person.” Some special circumstances allow guest sharing, for example, children under the age of 14 can live with their parents, and elderly people without the ability to live independently can live with their guardians.

### Medical Observation

Medical observation is an effective tool to control the spread of infectious diseases. In general to minimize contact, medical staff should inquire about the physical condition of the quarantined personnel through telephone or a social App on a daily basis, such as whether there is a cough, fever, dyspnea, etc., but special care should be provided to certain guests (Table 1).

**Table 1.** Give special care to special groups

Special groups	Special care
Close contacts	Face to face temperature test, Check for respiratory symptoms
Mental patient	Face to face temperature test, pay attention to mental state, give more care, arrange psychological consultation
Pregnant woman	Give priority to food delivery, meet reasonable requirements
The aged	Give priority to food delivery, meet reasonable requirements
Room with a baby	Avoid phone calls that scare babies
Severe chronic diseases patient	Necessary physical examinations, such as measuring blood pressure and blood glucose, should be given more care to ensure the supply of chronic medicines.

After March 22, all guests underwent a nucleic acid test for COVID-19 at the airport when they enter customs. The results usually became available during the quarantine period, and the medical staffs were informed through the liaison office. Those who have positive nucleic acid results or who develop respiratory symptoms or fever during isolation were sent by ambulance to designated hospitals or fever clinics for further diagnosis and treatment.

A fever clinic is often the first choice, for the severe acute respiratory syndrome (SARS) that occurred in China in 2002 to effectively control the source of infection, cut off the route of transmission, and prevent cross-infection and spread of the epidemic in the hospitals, many hospitals have gradually set up fever clinics in view of the characteristic that fever is the first symptom of SARS patients in most cases. All stable fever patients without emergency circumstances were first triaged to a fever clinic to rule out infectious diseases. In the fever clinic, epidemiological investigation and blood routine examination were conducted. Patients with high suspicion of infectious diseases were tested for the pathogen of the relevant infectious diseases.^6^

Patients who transferred from the isolation site to the fever clinic will undergo routine blood tests, chest CT, influenza A, influenza B, and COVID-19 nucleic acid tests.

If the results of further tests indicate that COVID-19 or other infectious diseases are not present, the patient will be returned to the isolation site upon completion of the necessary treatment and keep the state isolated until the end of the 14-d period. Confirmed patients get treatment at designated hospitals until they recover.

### Food Supplies

The hotel is equipped with a kitchen where guests can order food by phone or by means of the ordering App, and the food is delivered to the room by hotel staff at regular intervals. Cigarettes and alcohol are banned for safety and health reasons. Daily necessities can be delivered to the hotel reception desk by express delivery and then disinfected and distributed by the staff. Due to the shortage of staff, unless there are special circumstances, it is stipulated that each person can only have 1 chance to use express delivery once a week. Having inadequate basic supplies (eg, food, water, clothes, or accommodations) during quarantine is a source of frustration.^7^ Staff should ensure that adequate supplies are available to meet the guest’s basic needs, and it is important that they can be provided as quickly as possible.

### Psychological Health

Several studies have found that isolation can cause psychological problems, such as posttraumatic stress symptoms, avoidance, and anger.^8–10^ Many participants showed fear of infection, fear of catching it themselves, or fear of infecting others.^11–13^ In our experiences, some guests constantly disinfected their rooms and asked themselves if they are infected. Due to the absence of daily activities, the need for mental health care increased because of boredom, depression, isolation, and other psychological symptoms during the pandemic. Failure to provide specific requirements (such as cigarettes or alcohol) is often a source of frustration, and a lack of information can also be a source of stress.^14,15^ Sufficient attentions should be addressed to psychological problems, especially among those with mental illness. If possible, a psychologist can be appointed and mental health diagnostic tools can be used to help identify patients for treatment.^16^ The staff should regularly and supportively monitor well-being and psychosocial status of the guests to identify risks, emerging issues, and adaptively respond to their needs. Furthermore, encourage communication and speak openly about their concerns with trusted nurses or friends to reduce their anxiety.

### Release From Quarantine

All guests who have been quarantined for 14 d will receive a certification issued by the government that allows them to pass through all parts of mainland China and get back to their normal life.

## Discussion

While China has seen a significant decline of COVID-19 cases after making all-out efforts for more than 3 mo, the world is currently facing an escalating urgency, as the disease has broken out in multiple places and spread to more countries around the world. To control the global spread of the COVID-19, at present, many countries and cities have enacted strict measures, including closing borders, restricting movement, suspending public gatherings, and even imposing a lockdown on the whole country. These measures could prevent hundreds of thousands of infections.^17^ The epidemic situation is still severe beyond the border, putting the country under great challenge of imported cases. China has made all-out efforts to suppress the pandemic, for instance, nonpharmaceutical interventions including intercity travel restrictions, early identification, and isolation of cases, human contact restrictions and social distancing have effectively controlled the development of the epidemic.^18^ Some scholars found that only stricter quarantine measures can curb the outbreak of the disease^19^ and the separating public health function during an emergency helps correctly triage and ultimately route those affected.^20^

Most countries recommend quarantine for travelers returning from overseas affected areas, but usually only ask for self-quarantine. There is also no specialized agency to manage and assist travelers in transit and quarantine. Take the British government’s policy of travelers as an example,^21^ when you arrive in the United Kingdom, you will not be allowed to leave the place where you’re staying for the first 14 d you are in the United Kingdom unless you are arriving from an exempt country. You can take any form of transport to your quarantine accommodation including your own home, staying with friends or family, hotels, or other temporary accommodation. When you have no other choice, you can use public transportation to get to your destination. These lenient policies pose a potential risk that asymptomatic patients may transmit the virus to fellow passengers or to family and friends.

In China, only a few airports, such as Shanghai, Beijing, and Guangzhou, accept travelers returning from abroad, many of whom do not travel to those cities as destination and are not eligible for home quarantine. If travelers are allowed to leave on their own, it could trigger another outbreak. Therefore, the use of specialized vehicles to transport passengers and the setting up of centralized isolation sites for the use of passengers who do not have conditions for home isolation, through closed-loop management, may minimize the risk of further outbreaks. We believe that our isolation site measures not only apply to China, but also can provide a positive role for similar regions in reducing the spread of the virus.

China has suffered an economic strike from COVID-19 during the past 3 mo or so; those strict measures may lead to a sharp increase in unemployment, the uncontrolled outbreak leaves people with no choice. Nevertheless, with the progress of the measures, we expect to bring the epidemic under control and people will be able to resume their work and routine life.

As Shanghai is one of the economic centers of China as well as a port city, a large number of people pour into Shanghai from abroad, and the trend of the disease showed that imported cases began to appear and increased rapidly after March 5, which could cause the disease to erupt again.

To avoid that, Shanghai implemented strict measures, such as the establishment of a closed loop of a dedicated channel, and passengers with fever or suspected symptoms will be transferred to the designated medical institutions within 10 min by emergency vehicles. The asymptomatic travelers will be quarantined centrally, thus providing a positive contribution to prevent the re-outbreak of COVID-19.

To more quickly resume work and economic production, we strongly suggest that the stable areas, especially the port city, should adopt strict quarantine measures for people returning from affected areas.

In the process of setting up isolation sites, there are some key issues that should be addressed, such as the conflicts caused by immature team cooperation, confusion caused by unclear division of work, increased risk of infection caused by inadequate preparation and training of PPE, and physical and mental health problems caused by staff working in a closed environment for a long time.

Here are some tips on how to solve the above problems effectively:

(i) The government plays the leading role. Any measure that can be effectively implemented requires strong government leadership. Government departments have more administrative resources and power of approval than ordinary institutions; hence, the government also has more obligations. Public health leaders also should strengthen systems to provide oversight, control, and managerial capacity for more clear objectives while continuing informatics modernization.^22^
(ii) Unity and cooperation. The work at the isolation sites requires multisectoral cooperation. Unity and cooperation are our most potent weapons and only through strengthening our cooperation endeavors can we safeguard the health and well-being of the community.
(iii) Information collection and sharing. Only by active involvement in COVID-19 data sharing to provide timely, comprehensive, and transparent information can we avoid the occurrence of untraceable patients. This is especially important for tracking close contacts and preventing the spread of the virus.
(iv) Providing proper PPE to avoid infection of staff. Once a worker is infected, this isolation site must be shut down.
(v) We need a regular rotation of staff, in terms of improving their work performance and boost energy, usually once a month.

### Limitations

Although these measures can play a positive role in preventing the re-outbreak of the epidemic, it is also a complex task and a huge system that requires a lot of manpower, material resources, and financial resources. In addition, if the number of people needing centralized isolation is too large, it is also a challenge to establish enough centralized isolation sites quickly in a short period of time, especially in cities that do not have enough appropriate hotels. The data on the number of sites and admissions to those sites are also limited. As far as the authors know, the number of sites in Shanghai is changing dynamically according to the number of people needing centralized isolation.
